# Editorial: Bioengineering and biotechnology approaches in cardiovascular regenerative medicine, volume II

**DOI:** 10.3389/fbioe.2024.1380646

**Published:** 2024-02-16

**Authors:** Mehdi Salar Amoli, Zhen Ma, Yuji Nakada, Keiichi Fukuda, Jianyi Zhang, Vahid Serpooshan

**Affiliations:** ^1^ Wallace H. Coulter Department of Biomedical Engineering, Emory University School of Medicine and Georgia Institute of Technology, Atlanta, GA, United States; ^2^ Department of Biomedical and Chemical Engineering, Syracuse University, Syracuse, NY, United States; ^3^ BioInspired Syracuse Institute for Material and Living Systems, Syracuse University, Syracuse, NY, United States; ^4^ Department of Biomedical Engineering, School of Medicine and School of Engineering, University of Alabama at Birmingham, Birmingham, AL, United States; ^5^ Department of Cardiology, Keio University School of Medicine, Tokyo, Japan; ^6^ Department of Medicine, School of Medicine, University of Alabama at Birmingham, Birmingham, AL, United States; ^7^ Department of Pediatrics, Emory University School of Medicine, Atlanta, GA, United States; ^8^ Children’s Healthcare of Atlanta, Atlanta, GA, United States

**Keywords:** cardiovasclar disease, tissue engineering, regenerative medecine, stem cells, myocardial infarction

Recent advances in the treatment of cardiovascular diseases (CVDs) have come a long way towards addressing these complications and have significantly improved patient survival and quality of life. However, CVDs remain the leading cause of mortality and morbidity worldwide (Roth et al., 2020). This is partly due to the limited ability of mammalian heart tissue to regenerate itself after injuries (Kikuchi and Poss, 2012). Thus, significant research effort has been dedicated to understanding the underlying mechanisms of cardiac regeneration and developing strategies to enhance these processes. Among various techniques, cardiovascular tissue engineering and biofabrication strategies, aiming at creation of functional tissue analogues, have shown great promise. Given the critical importance of such efforts, this Research Topic presents several outstanding studies on the advancement and implementation of bioengineering and biotechnology principles in cardiovascular regenerative medicine.

The efforts to regenerate cardiac tissue requires integration of a range of disciplines, including basic biology of heart regeneration, cellular mechanisms governing cell proliferation and differentiation, plus advanced tissue engineering techniques, such as 3D organoid systems and biomaterials. Aiming to understand the mechanisms governing proliferative capacity of cardiomyocytes (CMs), Nguyen et al. employed single nucleus RNA sequencing to determine the parameters affecting CM cell cycle activation, resulting in a higher proliferative capacity of CMs in a pig model of myocardial infarction (MI) (Nguyen et al.). Different groups of molecules were identified to contribute to CM proliferation, paving the way for further studies including in human subjects. In a different study, this group also used single-cell RNA sequencing to examine another aspect of myocardial regeneration, the coronary vasculature. The single-nucleus RNA sequencing results demonstrated that only one subpopulation of endothelial cells (ECs) showed increased proliferation in response to the MI, while other EC population underwent endothelial-to-mesenchymal (EndMT) transition in the regenerating pig hearts. The latter EC population could be further examined in future studies as a potential new target to enhance heart regeneration.

A paramount factor to consider in devising cardiac regenerative strategies is the source of cardiac cells, such as CMs and cardiac fibroblasts. To date, induced pluripotent stem cell (iPSC)-derived CMs are increasingly employed in a variety of cardiovascular tissue engineering efforts. Wang et al., examined the impact of somatic-cell lineage of iPSCs on their CM differentiation by comparing the iPSCs derived from cardiac (atrial and ventricular fibroblasts) and non-cardiac (skin, kidney, and peripheral blood) lineage cells (Wang et al.). The cardiac origin iPSCs showed no significant differences in CM differentiation yield, and the resulting CMs exhibited consistent expression of pluripotency genes and electrophysiological function. However, these features altered significantly when comparing cardiac versus non-cardiac originated iPSC-CMs, signifying the importance of the iPSC origin in cardiac regenerative therapies. Another study focusing on the importance of cell origin, by Floy et al., investigated the differences between valve interstitial fibroblasts and left ventricular cardiac fibroblasts (LVCFBs) (Floy et al.). Transcriptomic analysis demonstrated a range of genes that differed in their expression among the two groups, suggesting LVCFBs to be more susceptible to conversion to myofibroblasts. Subsequent analyses of calcification and ECM synthesis demonstrated critical differences between the 2 cells, highlighting the importance of cell selection for regenerative strategies.

In parallel to the rapidly growing applications of iPSC-CMs *in vivo* regenerative therapies, these cells offer an unprecedented opportunity for the creation of robust *in vitro* models to study cardiac homeostasis and diseases. In a study by Shi et al., an *in vitro* iPSC-CM model was employed to gain a deeper insight into the cardiac muscle mechanobiology by examining the assembly and maturation of costameres, a structural-functional component of striated muscle cell, and their impact on myofibrillogenesis (Shi et al.). It was shown that the formation of costameres and myofibrils took place in an alternating fashion. The presence of pre-costameres affected myofibrilla formation, which in turn led to maturation of costameres. It was also shown that costamer maturation was impacted by contractile power of CMs, highlighting the importance of the extracellular matrix (ECM) mechanical properties, such as stiffness, on cardiac physiological functions. Stem cell-based *in vitro* platforms have also found increasing applications in disease modeling. Wang et al., reviewed recent efforts on establishing stem cell-based models of human fibrosis which engages a wide spectrum of organs, including the cardiovascular system (Wang et al.). This article provides a comprehensive and updated summary of different strategies that have been developed and utilized for accurate modeling of fibrotic pathologies.

In addition to the different cell sources, the bioengineering technologies applied can also have a significant impact on the success of cardiac regenerative therapies. Organoid technology, for instance, has found numerous applications in the field, as they provide a three-dimensional (3D) cellular microenvironment, replicating certain structural and functionalities of the native heart tissue. However, certain aspects of the organoid/spheroid systems, such as vascularization, pose challenges towards accurate representation of the cardiovascular tissues. Liang et al., reported the significance of Wnt signaling modulation as a method to develop vascularized cardiac organoids containing CMs and ECs (Liang et al.). Treatment with a range of small molecules, enabling codifferentiation of PSCs into CMs and cardiac ECs resulted in formation of vascularized organoids that can serve as a model for drug toxicity analyses. Similarly, aiming to generate an accurate replicate of cardiac tissue, Kahn-Krell et al., presented a facile approach to generate spheroids containing four major iPSC-derived cardiac cell types, including CMs, ECs, smooth muscle cells, and cardiac fibroblasts (Kahn-Krell et al.). These spheroids, created via a 3D suspension culture method, expressed major functional cardiac markers, and further established a robust *in vitro* platform for further research in cardiac regeneration. Considering the importance of vascularization in organoid development aimed at regeneration of complex tissues, LaMontagne et al., reviewed strategies to vascularize cortical organoids (LaMontagne et al.). One promising technique incorporates ECs along with signaling molecules into the organoids to induce vessel formation. Fusion of vascular spheroids with cortical organoids, and codifferentiation of different cell types are other robust strategies. Replication of native ECM cues, such as mechanical properties or proteins like collagen, is also known to impact angiogenesis. Further, bioreactors that enable dynamic flow surrounding the spheroids are also suggested as a method to enhance vascularization. These approaches could be applied to the cardiac regeneration field enhancing the ability of developed spheroids in recapitulating the native tissue structures.

Over the past 2 decades, a variety of bioengineering methods have been employed to amplify the regenerative capacity of endogenous or exogenous CMs. A study by Xiao et al., reviewed different approaches employed for enhancing the therapeutic impact of CMs derived from pluripotent stem cells (Xiao et al.). These strategies, including pre-treatment of cells with biomolecules such as growth factors, modifying the gene expression profile to enhance proliferative capacity of CMs, and co-transplantation with other cell types such as ECs or mesenchymal stem cells can have a significant impact on the outcome of cardiac tissue engineering strategies. In addition to the cellular methods described above, biomaterial-based strategies are a major area of research in cardiac regenerative strategies. Guo et al., described current advances in hemostatic biomaterials, including naturally derived biomaterials like protein- or polysaccharide-based materials, or inorganic biomaterials, such as zeolite and kaolin (Guo et al.). Description of hemostatic biomaterial mechanism of action, including physical, chemical, and physiological mechanisms, and further suggestions on design of these biomaterials demonstrate a clear path for future research.

Among biomaterial-based cardiac tissue engineering methods, decellularized ECM (dECM) strategies have gained increasing attention both for basic science research, as well as translational applications. Lopera Higuita et al., used decellularized bovine vein ECM as scaffolds for development of vascular grafts (Lopera Higuita et al.). Using an antigen removal processing method, they circumvented some key disadvantages of other decellularization techniques, maintaining the structure and composition of the natural ECM. The team demonstrated superior EC proliferation and migration on the scaffolds due to maintenance of basement membrane proteins. In another dECM-based approach, Guo et al., evaluated the functional impairment of tissue-engineered blood vessels (TEBVs) due to the inflammatory response (Guo et al.). Decellularized carotid arteries of female pigs were coated with anti-CD34 antibody functionalized heparin-collagen. The team demonstrated the effect of intravenous transplantation of regulatory T cells in inhibition of intimal hyperplasia. Adverse remodeling of vascular grafts is the focus of another study in this Research Topic, by Tan et al., where a wide range of strategies are suggested to prevent such processes (Tan et al.). These include cell-based strategies, such as iPSC incorporation, or biomaterial-based strategies, such as scaffold functionalization.

In addition to the applications exemplified above, 3D scaffold systems are also increasingly used for delivery of biomolecules/drugs to enhance tissue regeneration. Clayton et al., developed plasma polymerized nanoparticles and demonstrated their use for delivery of platelet derived growth factors to cardiac cells both *in vitro* and *in vivo* (Clayton et al.). The nanoparticles did not pose any adverse effect on viability or contractile function of CMs, while maintained the functionality of conjugated growth factors. This method for direct delivery of growth factors can boost the regenerative capacity of various tissue engineered implants in the future.

In summary, the unprecedented advances in the 21st century, in the fields of cardiovascular bioengineering and regenerative medicine have enabled development of robust platforms for both *in vitro* disease modeling and drug screening, as well a variety of clinical therapies. Some key future directions and grand challenges at the interface of engineering and cardiovascular medicine include personalized and precision medicine, cell and genome engineering, and the design and development of smart biomedical devices.
